# Tailored internet-based psychological treatment for psychological problems during the COVID-19 pandemic: A randomized controlled trial

**DOI:** 10.1016/j.invent.2023.100662

**Published:** 2023-08-25

**Authors:** Victoria Aminoff, Johanna Bobeck, Sofia Hjort, Elise Sörliden, Mikael Ludvigsson, Matilda Berg, Gerhard Andersson

**Affiliations:** a Department of Behavioural Sciences and Learning, Linköping University, Linköping, Sweden; b Department of Biomedical and Clinical Sciences, Linköping University, Linköping, Sweden; c Department of Psychiatry, Linköping University, Linköping, Sweden; d Department of Acute Internal Medicine and Geriatrics in Linköping, Linköping University, Linköping, Sweden; e Department of Health, Medicine and Caring Sciences, Linköping University, Linköping, Sweden; f Department of Clinical Neuroscience, Karolinska Institute, Stockholm, Sweden

**Keywords:** Internet-based cognitive behavior therapy, COVID-19, Depression, Psychological treatment, Randomized controlled trial

## Abstract

The COVID-19 pandemic influence mental health in both infected and non-infected populations. In this study we examined if individually tailored internet-based cognitive behavioral therapy (ICBT) could be an effective treatment for psychological symptoms related to the pandemic. Following recruitment we included 76 participants who were randomized to either a treatment group (*n* = 37) or a waitlist control group (*n* = 39). The treatment group received 8 modules (out of 16 possible) during 8 weeks with weekly therapist support. We collected data on symptoms of depression, experienced quality of life, anxiety, stress, anger, insomnia, PTSD, and alcohol use before, after the treatment and at one year follow-up. Using multiple regression analysis, group condition was found to be a statistically significant predictor for a decrease, favoring the treatment group, in symptoms of depression, insomnia, and anger with small to moderate effect sizes. The improvements remained at one year follow-up. Group condition did not significantly predict changing symptoms regarding experienced quality of life, anxiety, stress, PTSD and alcohol use. Findings indicate that ICBT is an effective intervention for some psychological symptoms associated with the COVID-19 pandemic. There is a need for further studies on mechanisms of change and on tailored ICBT for problems associated with crises like the pandemic.

## Highlights

• This is a randomized controlled trial aiming to investigate individually tailored ICBT during the COVID-19 pandemic.
• The intervention was effective in reducing symptoms of depression, insomnia and anger, in comparison to a wait-list.
• The intervention was not significantly effective for anxiety, post-traumatic stress, stress or alcohol consumption.
• Overall, ICBT seems to be effective for some psychological symptoms during the COVID-19 pandemic.

## 1 Introduction

The COVID-19 pandemic, caused by the SARS-CoV-2 virus, has been associated with mental health problems, such as post-traumatic stress symptoms, anger, confusion (Brooks et al., 2020), as well as health anxiety (Kurcer et al., 2021). The need of physical, and thus to some extent social, distancing and quarantine has led to changes in behavioral patterns (Galea et al., 2020), which has been vital to mitigate the spread of the SARS-CoV-2 virus. A problem arising, though, is that longer quarantine time, fear of infection, boredom, inadequate information, financial loss and stigma have been found to be associated with more distress (Brooks et al., 2020).

Cognitive behavior therapy (CBT), with broad scientific support from randomized controlled trails and meta-analyses, is widely used for several mental health problems and disorders such as depression and anxiety (Hofmann et al., 2012). CBT consists of a number of interventions that combine behavioral, cognitive and emotion-focused techniques. For further information about the theory behind CBT, please see e.g. Hofmann (2011). One way to deliver CBT is to use the internet (ICBT), which is an effective treatment option for several psychiatric disorders and symptoms, such as depression, generalized anxiety disorder, PTSD, panic disorder and social anxiety disorder (Andersson et al., 2019a). ICBT targeting somatic health problems, for example chronic pain and tinnitus has also shown to be effective (Mehta et al., 2019).

When including support or guidance by a therapist, ICBT tends to be comparably effective to CBT face-to-face for depression and anxiety symptoms (Hedman-Lagerlöf et al., 2023). When compared with no therapist support, ICBT with guidance has been found to be advantageous for symptom reduction (Andersson et al., 2019b). This has also shown to be the case during the COVID-19 pandemic (Oehler et al., 2021). ICBT can be a manualized and set up for a specific diagnosis or a set of transdiagnostic symptoms, or be individually tailored to the individual participant's current symptoms and experienced problems (Andersson, 2018), which facilitates addressing comorbidity (Johansson et al., 2012). The effects of ICBT have shown to be long-lasting, with follow-up studies even longer than two years, for depression, generalized anxiety disorder, panic disorder and stress for example (Andersson et al., 2018).

One additional advantage using ICBT for treatment delivery, in relation to the COVID-19 pandemic, is that no physical meetings between the therapist and the patient are required. The COVID-19 pandemic affected how the provision of mental health services adequately should be executed (Korecka et al., 2020). Mahoney et al. (2021) reported a five time increase in number of people registered for ICBT between April and June 2020 compared to the same period the year before, which illustrates a cumulative need for psychological interventions via internet. The effects of ICBT were shown to be similar during these periods both years (Mahoney et al., 2021).

ICBT has shown promising results in treating depression and anxiety during the COVID-19 pandemic but needs to be investigated further (Komariah et al., 2022). With randomized controlled trials (RCT), psychological interventions with different content for people with confirmed SARS-CoV-2 infection have been investigated (Liu et al., 2020; Sotoudeh et al., 2020; Wei et al., 2020). The results are promising regarding symptoms of depression (Wei et al., 2020) and anxiety (Liu et al., 2020) among others. Regarding the general population and people who were not necessarily infected by the SARS-CoV-2 but still experienced psychological symptoms, Al-Alawi et al. (2021) found that six weeks of ICBT had positive effects on depression and anxiety symptoms. In another RCT, Wahlund et al. (2021) investigated a three-week unguided ICBT intervention addressing dysfunctional worry in relation to the COVID-19 pandemic, with good results regarding both symptoms of worry and other outcomes such as insomnia. Perri et al. (2021) did a controlled study and reported that depressive and anxiety symptoms related to the pandemic during quarantine, isolation or work in COVID-19 hospital wards were reduced in both the group who received ICBT and the group who received internet-based eye movement desensitization and reprocessing (EMDR). Also, unguided ICBT delivered through a mobile app during the COVID-19 pandemic was found to have effects on depression and insomnia symptoms (Song et al., 2021). However, in one RCT by Brog et al. (2022), no significant treatment effects on depression, anxiety and stress symptoms were reported when they evaluated ICBT for psychological symptoms related to the COVID-19 pandemic. Small effects on emotion regulation skills and resilience were however found. In sum, the results of ICBT for psychological symptoms during the COVID-19 pandemic are promising but more research is needed.

Our research group conducted an early pilot study comparing seven weeks of tailored guided ICBT against a wait list control group (Aminoff et al., 2021). As we by that stage did not know what symptoms and problems people in the general population would experience, we included 16 possible modules (i.e. treatment modules within the ICBT program) to select from. The results from the pilot study (*N* = 52), conducted in the summer of 2020 in Sweden revealed significant symptom reductions in favor for the treatment group, including measures of depression, anxiety, and stress symptoms, with moderate to large between-group effect sizes.

To further explore the results from the pilot study (Aminoff et al., 2021), and to investigate the effects of ICBT for psychological symptoms related to the COVID-19 during the second wave of the pandemic (spring 2021), this study evaluated the ICBT program in a controlled trial. The treatment lasted for eight weeks and was individually tailored with eight prescribed treatment modules guided by a therapist on a weekly basis. More specifically, our aim was to explore whether individually tailored ICBT could be effective to reduce symptoms of depression and increase experienced life quality for people with psychological symptoms related to the COVID-19 pandemic. We also included secondary outcomes and a one-year follow-up. By the time this trial was conducted, it was still uncertain how people were affected by the COVID-19 pandemic. Thus, the secondary outcomes (described below) were aimed to be broad within an exploratory approach and are reused from the pilot study (Aminoff et al., 2021) for the opportunity to compare the two studies with each other.

## 2 Method

### 2.1 Trial design

Included participants were randomized on a 1:1 ratio to either a treatment group or a control group by a person not involved in the research. In the treatment group participants received guided ICBT for eight weeks with therapist support once a week through a secure platform (Vlaescu et al., 2016). The control group participants were assigned to a waitlist. Pre-treatment and post-treatment measures, consisting of primary and secondary measures described below, were administered before and after the eight-week treatment period. The control group received treatment after post-treatment measures had been collected and post-treatment interviews finished. The study was a continuation of our pilot study (Aminoff et al., 2021) with some minor changes. The study protocol was approved by The Swedish National Ethics Committee (Dnr 2020–02313) and is registered on ClinicalTrials.org (NCT04424212). A one-year follow-up measurement was also included.

### 2.2 Recruitment and participants

The recruitment phase was initiated in January 2021 and continued for two weeks. Power analysis was based on a power of 0.80 and an expected effect size of Cohen's *d* = 0.70 on the primary outcomes, which resulted in a sample size of 120 participants with a 1:1 ratio. Considering possible dropout rate, we aimed to recruit 160 participants. Advertising was made on social media and in a national newspaper. All ads consisted of brief information about the study and its purpose, directing individuals to the study's website www.coronacope.se. On the website, further information about the study was provided and interested individuals could register by signing an online informed consent and fill in the pre-treatment measures. If an individual fulfilled the initial inclusion criteria, a semi-structured clinical interview was scheduled administered by telephone within a few days. The interview consisted of questions about the individual's core reasons for participating in the study and open-ended questions about mental and physical health problems. The aim was to gain further information about treatment suitability and how to tailor the treatment to each individual's specific needs. Potential obstacles for participating in the study and completing the treatment were also considered based on the interview. Furthermore, for security reasons, the interview included an assessment of suicide risk. After the interview, eligible participants' data were discussed during case management meetings with the researchers and decisions about inclusion or exclusion were made. Following inclusion, potential modules suitable for the specific participants' needs were discussed during the meetings. If risk of suicidality was judged as being too high, the individual was excluded and informed by telephone about how to seek other health care (which in Sweden does not demand special insurance since it is tax-funded and potential contact with the study psychiatrist could be offered). Individuals excluded for other reasons, such as having no significant problems, more severe psychiatric problems, ongoing treatments or having problems not addressed in the treatment material, received a personalized email with information about exclusion and the reasons why.

**Fig. 1:**
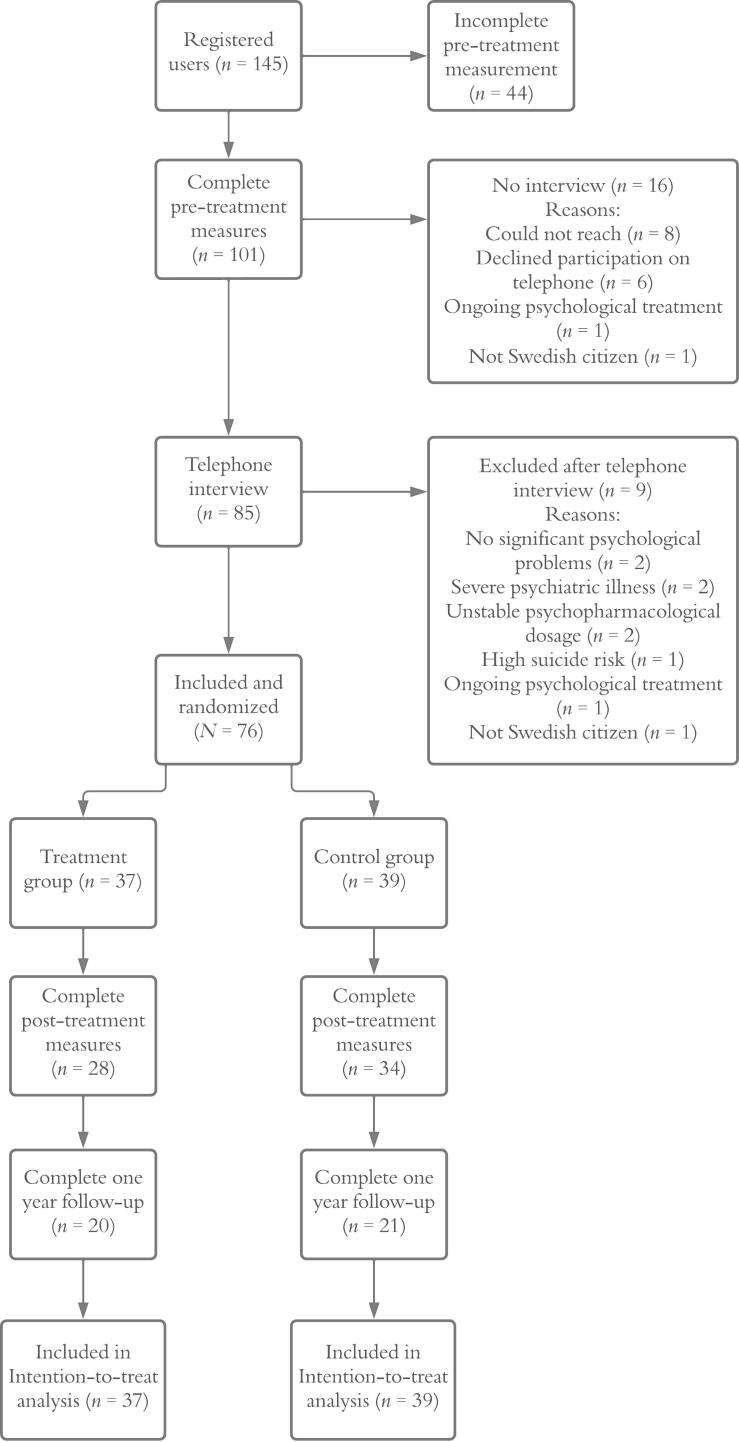
Flow chart of the study recruitment process.

Altogether, 101 individuals completed the pre-treatment measures and were contacted for a telephone interview. After excluding 25 individuals (see Fig. 1) a total of 76 participants were included.

We asked questions about COVID-19 status and history. Nine participants (11.8 %) stated that they had been tested positive for COVID-19, 65 persons (85.5 %) had not and 2 (2.6 %) were uncertain. When asked about typical COVID-19 symptoms (e.g., loss of smell, breathing problems), 33 (43.4 %) reported that they had experienced such symptoms, 32 (42.1 %) had not and 11 (14.5 %) were uncertain. Further descriptive statistics for the included participants are presented in Table 1. Participants were randomized to treatment (*n* = 37) or to control group (*n* = 39) using an online random number generator (www.random.org), performed by a person not involved in the study.

**Table 1:** Descriptive statistics of the included participants at pre-treatment assessment, *N* = 76.

Baseline characteristics	Treatment (*n* = 37) *n* (%)	Control (*n* = 39) *n* (%)	Total (*N* = 76) *n* (%)
Age			
Mean (SD) years	33.8 (13.7)	37.6 (17.9)	35.7 (16.0)
Range	22–74	21–83	21–83

Gender			
Female	34 (91.9)	36 (92.3)	70 (92.1)
Male	3 (8.1)	3 (7.7)	6 (7.9)

Highest educational level			
Nine year –compulsory school	0 (0)	0 (0)	0 (0)
Secondary school	1 (2.7)	2 (5.1)	3 (3.9)
Vocational school	2 (5.4)	3 (7.7)	5 (6.6)
College/university -----(not completed)	14 (37.8)	11 (28.2)	25 (32.9)
College/university -----(completed)	20 (54.1)	23 (59.0)	43 (56.6)
Other	0 (0)	0 (0)	0 (0)

Occupational status			
Student	14 (37.8)	10 (25.6)	24 (31.6)
Employed	17 (45.9)	19 (48.7)	36 (47.4)
Unemployed	3 (8.1)	4 (10.3)	7 (9.2)
Retired	2 (5.4)	4 (10.3)	7 (9.2)
Parental leave	1 (2.7)	1 (2.6)	2 (2.6)
Sick leave	0 (0)	1 (2.6)	1 (1.3)
Other	0 (0)	0 (0)	0 (0)

Experience of psychological treatment			
None	14 (25.8)	10 (25.6)	24 (31.6)
Previously	21 (56.8)	27 (69.2)	48 (63.2)
Ongoing	2 (5.4)	2 (5.1)	4 (5.3)

Psychopharmacological medication			
None	29 (78.4)	25 (64.1)	54 (71.1)
Earlier	4 (10.8)	4 (10.3)	8 (10.5)
Ongoing	4 (10.8)	10 (25.6)	14 (18.4)

### 2.3 Criteria for participation

Eligibility criteria were the following a) experience of psychological symptoms or problems connected to the COVID-19 pandemic, b) ability to speak, read and write Swedish, c) access to a smartphone, tablet, computer or another device with internet, d) 18 years or older. Experience of psychological symptoms or problems connected to the COVID-19 pandemic was assessed through an open-ended question in the pre-treatment measurement (“How does the COVID-19 pandemic affect your mental well-being today?”), the measure The Coronavirus Health Impact Survey (CRISIS; Nikolaidis et al., 2021), and the semi-structured clinical telephone interview. No cutoff scores were used.

The individual was excluded if any of the following criteria were met a) not being a Swedish citizen, b) severe psychological or somatic illness hindering participation c) ongoing alcohol or substance abuse d) high risk of suicide, e) ongoing psychological treatment interfering with the study treatment, and f) altered psychopharmacological medication dosage during the previous three months or a planning to alter such a dosage during the study period. Regarding high suicide risk, an overall assessment was made based on the pre-treatment measures and the telephone interview. For example, existing plans or more concrete thoughts about how and when to put the plan into practice were considered as high risk of suicide whereas merely thoughts about death or suicide were not. A few individuals had an ongoing psychological treatment contact but were still included in the study. In these cases the other ongoing treatment had a clearly different focus than our treatment (non-CBT oriented supportive counseling and also not recently started), and was seen as unlikely to influence participation of the study and its effects.

### 2.4 Treatment

The eight-week tailored treatment consisted of a selection of up to eight modules, including one introduction module (Introduction) and one closure/ending module (Maintenance) that all participants received. There were 14 other modules available to choose between for the tailoring of the treatment and each participant was assigned six additional tailored modules. All modules were based on CBT principles and each module had a specific focus: behavioral activation, negative thoughts, anxiety, worry, panic, social anxiety, emotions, acceptance, relaxation, sleeping problems, stress, perfectionism, problem-solving, and difficult memories. The aim of including these specific modules as possible to work with during the treatment was to give the participant a range of strategies and focus areas, in line with common forms of psychopathology. The modules have also been shown to be effective in earlier studies for depression (Johansson et al., 2012) and anxiety (Carlbring et al., 2011). With the exception for the two obligatory modules participants had the opportunity in the first module to wish which of the problem areas/modules they wanted to include in their remaining treatment. Based on their wishes together with the information from pre-treatment measures and telephone-interview, specific modules were then selected by the research group. The modules offered were the same as in the previous pilot study (Aminoff et al., 2021), with a few adaptions given the circumstances of the pandemic in Sweden at that time. All modules contained psychoeducation and exercises. For more detailed description of the treatment modules, see Appendix A.

Each participant was linked with a therapist. All communication between the participants and their therapist was done through a secure platform with two-step verification, in which distribution of modules and self-report measures also were managed (Vlaescu et al., 2016). The therapists provided online feedback on the assignments using text messages via the platform on a weekly basis. Additional support was given within 24 h when asked for by the participants. The study therapists were psychologist students in their final year of a five-year clinical psychology program. During the psychology program, the students undergo corresponding step one (of two) of the psychotherapy program and thus have both theoretical and practical CBT competencies in their final year. They were supervised by three clinical psychologists and the principal investigator on a weekly basis. A psychiatrist was also available for consultation throughout the study.

On a weekly basis the participants in both the treatment and the control group were asked to complete the Patient Health Questionnaire-9 (PHQ-9) to monitor any deterioration during the treatment. The control group were allowed to contact the study staff if needed during the whole study. The control group received the same treatment two weeks after the treatment group had finished their eight weeks of treatment, when post-treatment measures had been collected and short follow up-interviews by telephone had been administered.

### 2.5 Measures

All measures were administered at both pre- and post-treatment assessments, except for the The Coronavirus Health Impact Survey (CRISIS; Nikolaidis et al., 2021), which was administered only at pre-treatment. The measures used were the Beck Depression Inventory (BDI-II; Beck et al., 2005), Brunnsviken Brief Quality of Life (BBQ; Lindner et al., 2016), Patient Health Questionnaire-9 (PHQ-9; Kroenke et al., 2001), Generalized Anxiety Disorder-7 (GAD-7; Spitzer et al., 2006), Alcohol Use Disorder Identification Test (AUDIT; Berman et al., 2012), Insomnia Severity Index (ISI; Bastien et al., 2001), Impact of Events Scale-Revised (IES-R; Weiss and Marmar, 1997), Perceived Stress Scale (PSS-14; Cohen et al., 1983), and Dimensions of Anger Reactions (DAR-5; Goulart et al., 2021).

CRISIS is a self-report measure addressing various aspects of mental and somatic health, and risk and health factors in relation to the COVID-19 pandemic (Nikolaidis et al., 2021). It was used to assess how the individuals had been affected by the COVID-19 pandemic. When tested, CRISIS has shown to have excellent internal and test-retest reliability and Nikolaidis et al. (2021) also argued it to have a good construct validity. A couple of background questions, written by the research group, about experiences related to the pandemic were also included in the post-treatment measure, for example if the participants had been infected by the SARS-CoV-2 virus during the treatment.

#### 2.5.1 Primary outcome measures

Primary outcome measures in this trial were the BDI-II and BBQ. Internal consistency was calculated with Cronbach's alpha (α).

##### 2.5.1.1 BDI-II

The 21 items questionnaire BDI-II is commonly used for measuring symptoms of depression (Beck et al., 2005). It has shown excellent internal consistency (α = 0.92) and good test-retest reliability (*r* = 0.93; Beck et al., 2005). With a total score ranging from 0 to 63, the results 0–13 are interpreted as minimal depression, 14–19 as mild, 20–28 as moderate and 29–63 as severe depression (Beck et al., 2005). In this study, Cronbach's alpha for the BDI-II was 0.86.

##### 2.5.1.2 BBQ

This measure was developed to investigate experienced quality of life and it has demonstrated to have fair internal consistency (α = 0.76) and high test-retest reliability (ICC = 0.82) by Lindner et al. (2016). It consists of 12 statements in which the respondent is asked to estimate how well the statements matches the own experience from 0 (do not agree at all) to 4 (fully agree). The total, maximum score is 96, and a higher score implies a higher experienced quality of life (Lindner et al., 2016). The cutoff 52 has been shown to adequately distinguish a clinical group from and a non-clinical group (Lindner et al., 2016). In the present sample, Cronbach's alpha for the BBQ was 0.69.

#### 2.5.2 Secondary outcome measures

Secondary outcome measures included the PHQ-9, GAD-7, PSS-14, ISI, IES-R, DAR-5 and AUDIT. As with the primary outcome measures, internal consistency was calculated with Cronbach's alpha (α).

##### 2.5.2.1 PHQ-9

Like the BDI-II, the PHQ-9 was used to measure depressive symptoms, with higher scores reflecting more severe symptoms (Kroenke et al., 2001). Based on the respondents' answers on nine questions, the total score range between 0 and 36. The cutoff for mild depression is a score above four, for moderate depression a score above nine, for moderate to severe depression a score above 14 and for severe depression a score above 19 (Kroenke and Spitzer, 2002). The PHQ-9 had a Cronbach's alpha of 0.79 in the present study.

##### 2.5.2.2 GAD-7

The GAD-7 was included for assessing symptoms of worry and anxiety. GAD is scored from 0 to 21 and the total score can be interpreted as mild (5–10), moderate (11–15), or severe (above 15; Spitzer et al., 2006). In this sample, Cronbach's alpha for the GAD-7 was 0.87.

##### 2.5.2.3 PSS-14

To assess symptoms of stress, the PSS-14 (Cohen et al., 1983) was used. It consists of 14 items and the respondent rates how often he or she has experienced common symptoms of stress from never (0) to very often (4) the last month. This can result in a total score of 56 (Cohen et al., 1983). To our knowledge, there is no formal cutoff value defined for clinical significance on the PSS-14. In this study, the Cronbach's alpha of the PSS-14 was 0.82.

##### 2.5.2.4 ISI

The ISI is a 7-item questionnaire developed to assess symptoms of insomnia (Bastien et al., 2001). The items contain questions about experienced sleeping difficulties the last two weeks on a scale from none (0) to very severe (4). This gives a total score range between 0 and 28, and the clinical cutoff has been defined as a score of 10 (Bastien et al., 2001). Cronbach's alpha was 0.87 in this trial.

##### 2.5.2.5 IES-R

For exploring symptoms of post-traumatic stress disorder (PTSD), the IES-R (Weiss and Marmar, 1997) was administered. The measure address symptoms of re-experiencing, avoidance and hypervigilance and includes 22 items. Higher scores imply more severe symptoms of PTSD, however Weiss (2004) advocates not using any cutoff value for IES-R. The maximum total score is 88. In this study, Cronbach's alpha for the IES-R was 0.95.

##### 2.5.2.6 DAR-5

To assess experiences of felt anger, the DAR-5 (Goulart et al., 2021) was used. In this short 5-item self-report questionnaire, the respondent is asked to match their anger experiences on a scale from one (none or almost none of the time) to five (all or almost all the time). The total score can range from 5 to 25, with higher scores indicating feelings of anger to a greater extent (Goulart et al., 2021). The cutoff 12 is proposed for signaling psychological distress and impairment of function (Forbes et al., 2014). Cronbach's alpha for the DAR-5 in this trial was 0.85.

##### 2.5.2.7 AUDIT

The AUDIT was used to detect and assess alcohol use and misuse (Berman et al., 2012). It consists of 10 items and the maximum total score is 40. Cutoff score for risky and potentially harmful drinking is 6 for women and 8 for men (Berman et al., 2012). The AUDIT had a Cronbach's alpha of 0.75 in this sample.

### 2.6 Statistical analyses

All statistical analyses were calculated using IBM SPSS Statistics, Version 28. Results were considered as significant at the level of *p* < .05. Confidence intervals are reported at 95 %. To investigate differences between the treatment and control group *χ*^2^-test for categorical and *t*-test for continuous variables were used. These tests were also used for dropout analysis and to explore any differences between participants who did and did not complete post-treatment measures.

Results were calculated using an Intention-to-treat (ITT) approach (Complete Case Analysis results are available by request). Using multiple imputation, missing data at post-treatment measures were accounted for in the ITT, which included 20 imputations, as recommended by Enders (2017). Multiple imputation relies on the assumption that data are Missing at Random, i.e. a systematic relationship between the propensity of missing data and observed data is allowed where the missing data can be motivated depending on the observed data (Enders, 2017). To explore treatment effects, multiple regression analyses were performed, using forced entry, as recommended by Studenmund and Cassidy (1987). All predictors were added into the regression model simultaneously. Predictors used was group condition and the pre-treatment measure, as we wanted to control for the pre-treatment scores. Outcome variable was the post-treatment measure. Assumptions of the linear model, including normally distributed errors, independent residuals, homoscedasticity, additivity, and linearity, were checked. Regarding multiple regression, the assumptions of non-perfect multicollinearity were also checked. At the one year follow-up, paired samples *t*-tests were calculated with imputed data. This was done because there was no control group, as they had received the same as treatment the treatment group after post-treatment assessment.

Cohen's *d* was used for calculating and interpreting effect sizes, with the use of adjusted means of post-treatment measures, which were controlled for pre-treatment measures, and observed standard deviations at post-treatment measures. In line with recommendations by Cohen (1988), effect-sizes were interpreted as small (*d* = 0.20), medium (*d* = 0.50) and large (*d* = 0.80).

Reliable change index (RCI) was also calculated. RCI is a way to assess whether detected changes between pre-treatment and post-treatment measures are reliable or rather an effect of measurement error (Jacobson and Truax, 1991).

## 3 Results

When investigating the assumptions of the linear model, no outliers were detected.

Heteroscedasticity was explored with Levene's test. No violations were observed regarding linearity or independent residuals. This was explored visually through plots and investigated with Durbin Watson test, with all values between 1.605 (AUDIT) and 2.248 (IES-R). Calculating the variance inflation factor (VIF), no violations regarding the assumption of multicollinearity were detected.

### 3.1 Randomization check

No statistically significant differences were found between the treatment group and control group regarding age, gender, level of education, occupational status, earlier experiences of or ongoing psychological treatment or psychopharmacological medication (all *p*'s > 0.05). There were no statistically differences on the pre-treatment measures between the treatment and the control groups (all *p*'s > 0.05).

### 3.2 Missing data

A majority *n* = 62 (81.6 %) of the 76 included participants completed the post-treatment assessment. Two additional participants completed the BDI-II and PSS-14 at post-treatment, but did not finish the whole assessment battery, and were therefore categorized as non-completers of the assessment. Thus, the number of participants who did not complete the whole post-treatment measure was 14 (18.4 %), with nine (24.3 %) in the treatment group (*n* = 37) and five (12.9 %) in the control group (*n* = 39).

The treatment group and the control group did not differ in dropout rates investigated with *χ*^2^-test. No significant differences between completers and non-completers were found regarding gender, level of education, occupational status, age, or pre-treatment measures using *χ*^2^-test for the categorical variables, and *t*-test for the continuous variables (all *p*'s > 0.05). A non-significant result was shown when little MCAR's test was performed, *χ*^2^(2) = 20.12, *p* = .45, indicating that no obvious pattern exists in missing data.

### 3.3 Sample characteristics

As shown in Table 1, most of the in total 76 participants were female (92.1 %) and the mean age for the total sample was 35.74 (*SD* = 16.02). A majority had a college/university education (56.6 %) or underwent a college/university education (32.9 %). Table 2 shows observed pre-treatment and post-treatment data for all measures included in the trial.

**Table 2:** Observed means, standard deviations and number participants within the treatment group and the control group at pre- and post-treatment.

Measure and group condition	Pre-treatment measure			Post-treatment measure		
	M	SD	n	M	SD	n
Beck Depression Inventory-II						
Treatment	25.73	9.52	37	15.63	8.97	30
Control	27.05	8.72	39	23.03	10.52	34

Brunnsviken Brief Quality of Life						
Treatment	42.68	19.33	37	50.39	16.35	28
Control	35.13	18.26	39	39.79	15.89	34

Patient Health Questionnaire-9						
Treatment	11.65	5.65	37	7.71	4.43	28
Control	12.23	5.27	39	10.50	5.26	34

Genealized Anxiety Disorder-7						
Treatment	10.16	4.95	37	6.93	4.74	28
Control	10.77	4.54	39	8.97	4.59	34

Perceived Stress Scale-14						
Treatment	36.57	6.48	37	29.40	8.40	30
Control	37.64	6.14	39	34.12	7.19	34

Insomnia Severity Index						
Treatment	10.68	6.64	37	6.89	4.96	28
Control	12.92	6.16	39	10.94	6.35	34

Impact of Events Scale-Revised						
Treatment	31.81	22.88	37	23.64	17.85	28
Control	32.08	20.45	39	27.88	22.42	34

Dimensions of Anger Reactions-5						
Treatment	11.46	3.82	37	8.57	2.74	28
Control	11.05	4.41	39	10.32	3.58	34

Alcohol Use Disorder Identification Test						
Treatment	4.41	3.07	37	4.29	2.75	28
Control	4.38	4.31	39	4.32	4.26	34

### 3.4 Adherence and therapist time

On average, the participants in the treatment group opened 6.89 modules (SD = 2.35).

Number of completed modules, defined as having acquired an understanding of the main purpose of the module and expressing this in the exercises and in text-messages, was on average 4.14 (SD = 2.86). Using Pearson's *r*, no significant correlation between the number of opened modules and post-treatment outcome was found. Using Pearson's *r*, no significant correlation between the number of completed modules and change scores pre- and post-treatment was found (all *p*'s < 0.05).

The most common module that the participants in the treatment group completed was the Introduction, which 27 participants completed. Cognitive techniques and Behavioral activation were thereafter the second and third most common modules, with 21 and 18 participants completing them respectively. Other modules that were completed (by *n* participants in the parentheses) are as follows: Worry (14), Acceptance (13), Stress management (11), Maintenance (10), Sleep strategies (10), Relaxation (8), Emotion regulation (6), Perfectionism (6), Anxiety (5), Problem-solving (5), Difficult memories (4). No participant completed the modules Panic and Social anxiety.

Average therapist total time spent on the participants during the treatment was 132.6 min (SD = 76.39; range: 0 to 256 min), i.e. approximately 16.6 min per week.

### 3.5 Treatment outcome

Means and standard deviations for pre- and post-measures for the treatment group and the control group are displayed in Table 3.

**Table 3:** Pooled means, standard deviations, and number of participants with imputed data within the treatment group and the control group at pre- and post-treatment measure.

Measure and group condition	Pre-treatment measure			Post-treatment measure		
	M	SD	n	M	SD	n
Beck Depression Inventory-II						
Treatment	25.73	9.52	37	16.69	9.86	37
Control	27.05	8.72	39	22.87	11.15	39

Brunnsviken Brief Quality of Life						
Treatment	42.68	19.33	37	48.99	17.15	37
Control	35.13	18.26	39	39.82	17.81	39

Patient Health Questionnaire-9						
Treatment	11.65	5.65	37	8.12	4.83	37
Control	12.23	5.27	39	10.49	5.38	39

Genealized Anxiety Disorder-7						
Treatment	10.16	4.95	37	7.17	4.85	37
Control	10.77	4.54	39	8.92	4.68	39

Perceived Stress Scale-14						
Treatment	36.57	6.48	37	30.13	8.70	37
Control	37.64	6.14	39	34.14	7.53	39

Insomnia Severity Index						
Treatment	10.68	6.64	37	7.17	5.37	37
Control	12.92	6.16	39	11.03	6.23	39

Impact of Events Scale-Revised						
Treatment	31.81	22.88	37	24.41	18.62	37
Control	32.08	20.45	39	27.96	21.96	39

Dimensions of Anger Reactions-5						
Treatment	11.46	3.82	37	8.99	2.94	37
Control	11.05	4.41	39	10.27	3.61	39

Alcohol Use Disorder Identification Test						
Treatment	4.41	3.07	37	4.64	3.43	37
Control	4.38	4.31	39	4.43	4.49	39

Summary of unstandardized regression coefficients, standard error for these and standardized regression coefficients for group condition as predictor for each post-treatment measure are presented in Table 4. The pre-treatment measure was included as a predictor in each regression model. At post-treatment, a medium between group effect size was found for the BDI-II, *t*(73) = −2.44, *p* = .015, *d* = 0.51 95 % CI [0.05, 0.97], favoring treatment group in the decrease of depression symptoms. No significant effect at post-treatment was found on the BBQ, *t* (73) = 1.30, *p* = .195. Thus, one of the primary outcome measures seemed to significantly be predicted by group condition, whereas the other was not.

**Table 4:** Regression coefficients and *p*-value for group condition as predictor for post-treatment measure for each outcome variable, including pre-treatment measure also as a predictor.

Measure	Unstandardized coefficients		Standardized coefficients		
	B [95 % CI]	SE B	*β*	*t*	*p*
BDI-II	−5.41 [−9.765, −1.052]	2.22	−0.51	−2.44	0.015
BBQ	4.67 [−2.40, 11.74]	3.60	0.26	1.30	0.195
PHQ-9	−2.05 [−4.05, −0.06]	1.02	−0.39	−2.02	0.044
GAD-7	−1.38 [−3.29, 0.53]	0.97	−0.29	−1.42	0.156
PSS-14	−3.45 [−7.14, 0.24]	1.88	−0.42	−1.84	0.067
ISI	−2.68 [−5.15, −0.21]	1.26	−0.44	−2.13	0.034
IES-R	−3.41 [−11.68, 4.86]	4.22	−0.17	−0.81	0.419
DAR-5	−1.50 [−2.73, −0.27]	0.63	−0.45	−2.39	0.017
AUDIT	0.20 [−0.64, 1.04]	0.43	0.05	0.46	0.645

Note: BDI-II = Beck Depression Inventory-II, BBQ = Brunnsviken Brief Quality of Life, PHQ-9 = Patient Health Questionnaire-9, GAD-7 = Generalized Anxiety Disorder-7, PSS-14 = Perceived Stress Scale-14, ISI = Insomnia Severity Index, IES-R = Impact of Events Scale-Revised, DAR-5 = Dimensions of Anger Reactions, AUDIT = Alcohol Use Disorder Identification Test.

Regarding the secondary measures, group condition seemed to be a significant predictor of PHQ-9 scores at post-treatment measure, *t*(73) = −2.37, *p* = .033, with a small effect size favoring the treatment group, *d* = 0.39 95 % CI [0.07, 0.84]. Similar results were also found for the ISI, *t*(73) = −2.13, *p* = .034, *d* = 0.48 CI [0.02, 0.94], and the DAR-5, *t*(73) = −2.39, *p* = .017, *d* = 0.46 CI [0.01, 0.91].

No significant impact of group condition as predictor in the regression model, including pre-treatment measures as a predictor, was found for the GAD-7 (*t*(73) = −1.42, *p* = .156), the PSS-14 (*t*(73) = −1.835, *p* = .067), the IES-R (*t*(73) = −0.81, *p* = .419) and the AUDIT (*t*(73) = 0.46, *p* = .645).

### 3.6 Response and deterioration

Treatment response was assessed using RCI. RCI was calculated for the BDI-II. The standard deviation for the BDI-II (SD = 9.52) and the test-retest reliability reported by Beck et al. (2005) for BDI-II (*r* = 0.93) were used. RCI was calculated according to the equation described by Jacobson and Truax (1991). This resulted in a cutoff value of 6.98. Change values of the BDI-II between pre- and post-treatment above this value can be seen as reliable change and scores below −6.98 as reliable deterioration.

When calculating RCI we used the imputed data (ITT). Of the 37 participants included in the treatment group, 20 (54.1 %) showed a reliable change. Sixteen (43.2 %) did not reach reliable change or reliable deterioration. One participant (2.7 %) was classified as reliably deteriorated. Of the 39 participants in the control group, 15 (38.5 %) reached reliable change while 22 (56.4 %) did not. Two participants (5.1 %) in the control group were classified as reliably deteriorated.

### 3.7 One year follow-up

Based on an ITT approach, paired samples *t*-tests were used to investigate if the estimated symptoms at post-treatment had changed at one year follow-up. This was first performed for those measures with statistically significant treatment effects: BDI-II, PHQ-9, ISI, and DAR-5. The AUDIT was not included in the one year follow-up

BDI-II scores differed significantly between the post-treatment measure (*M* = 17.21, *SD* = 10.32) and at one year follow-up measure (*M* = 10.52, *SD* = 8.66), *t*(36) = 3.27, *p* = .001, with a moderate within-group effect size (*d* = 0.67, 95 % CI [0.30, 1.03]). With other words, the depression symptoms had decreased even more between the end of the treatment and one year later. The same was shown for the PHQ-9 between the post-treatment measure (*M* = 8.42, *SD* = 5.18) and the one year follow-up (*M* = 5.15, *SD* = 4.54), *t*(36) = 3.31, *p* = .001, *d* = 0.65 95 % CI [0.29, 1.00]. In contrast, no significant difference were shown for the ISI between post-treatment measure (*M* = 7.32, *SD* = 6.36) and the one year follow-up (*M* = 6.46, *SD* = 4.86), *t*(36) =0.769, *p* = .445. No significant differences were either found for the DAR-5 between the post-treatment measure (*M* = 8.96, *SD* = 3.01) and the one year follow-up (*M* = 8.99, *SD* = 2.89), *t*(36) = −0.048, *p* = .962. Thus, results show that the decrease in symptoms remained one year after treatment completion regarding symptoms of insomnia and anger.

When investigating symptoms at one year follow-up in comparison to the post-treatment measurement for the remaining measures, that were not found to differ in change between the treatment and the control group after the treatment, the BBQ was not found to differ, between the post-treatment measure (*M* = 49.34, *SD* = 16.94) and the one year follow-up (*M* = 55.33, *SD* = 20.21) with test statistics being *t*(36) = −1.541, *p* = .126. A significant difference was, though, found for the GAD-7 with a small effect size, *t*(36) = 2.04, *p* = .042, *d* = 0.39 95 % CI [0.05, 0.72], between the post-treatment measure (*M* = 7.25, *SD* = 4.83) and the one year follow-up (*M* = 5.06, *SD* = 3.73). This was also found for the IES-R, *t*(36) = 2.33, *p* = .022, *d* = 0.48 95 % CI [0.14, 0.82], between the post-treatment measure (*M* = 25.61, *SD* = 18.81) and the one year follow-up (*M* = 16.89, *SD* = 15.78). The post-treatment measure (*M* = 30.15, *SD* = 8.37) and the one year follow-up (M = 25.15, *SD* = 6.05) on the PSS-14 were found to differ with a moderate effect size, *t*(36) = 3.06, *p* = .003, *d* = 0.60 95 % CI [0.24, 0.94].

## 4 Discussion

The aim of this study was to investigate if ICBT could reduce psychological symptoms related to the COVID-19 pandemic and its consequences. The intervention showed promising effects as a way to treat psychological symptoms that had arisen or worsened during the COVID-19 pandemic, even if small or no effects were found on some measures. More specifically, there was a medium between group effect on the BDI-II (*d* = 0.51) and a small effect on the PHQ-9 (*d* = 0.39), both measuring symptoms of depression. We also found effects on the ISI (*d* = 0.48) measuring insomnia and on the DAR-5 (*d* = 0.46) measuring anger. However, we did not find statistically significant effects on quality of life as measured with the BBQ, anxiety measured with the GAD-7, stress symptoms measured with the PSS-14, post-traumatic stress symptoms measured with the IES-R or use of alcohol measured with the AUDIT. The decrease in symptoms on the ISI and the DAR-5 were maintained and even decreased more on the BDI-II and PHQ-9 at one year follow-up. Overall, the present study largely replicated our pilot trial findings (Aminoff et al., 2021) and indicate that ICBT can reduce some psychological symptoms associated with the COVID-19 pandemic. The differences in outcome between the studies were rather small with for example the results on stress symptoms (PSS-14) not reaching statistical significance. One obvious difference when comparing the studies is the time they were conducted, with the pilot study (Aminoff et al., 2021) being completed during summer of 2020 in the early phase of the pandemic and this trial during the winter/spring of 2021 when the pandemic was in a new wave. For example, many people's perceptions of the pandemic changed over that half year as social restrictions, economic effects and other aspects had changed in society.

Studies on the effect of ICBT targeting psychological symptoms related to the COVID-19 pandemic have generated different results. While some studies have reported effects on depression and anxiety symptoms (Al-Alawi et al., 2021; Wahlund et al., 2021; Wei et al., 2020), others have not (Brog et al., 2022). The treatments in the studies, including our own RCT, have had different contents, treatment duration, and levels of therapist support. These are examples of potential reasons for the different outcomes in addition to the time when the studies were conducted, and the samples included. This highlights the importance of defining for example the way and intensity of communication between the participant and therapist in a treatment study, which Seiferth et al. (2023) emphasizes in their guidelines. In the large literature on internet interventions there are clear indications that very brief treatments and treatments with minor or no involvement of a clinician tend to be less effective than treatments of similar length/contents as in face-to-face treatments (Andersson and Berger, 2012) or when guidance and support is included (Baumeister et al., 2014). With this study, we could examine the eight week long intervention, including weekly guidance by a therapist, and investigate whether these factors, among others, would be favorable also during the COVID-19 pandemic. This seemed to be the case for some psychological symptoms, but for others not.

More research is needed to investigate how a pandemic influences treatment uptake and effect as some treatment components like exposure in social anxiety disorder could not be implemented due to restrictions. In our two studies we used modules derived from our previous work on for example depression and anxiety, which include information and exercises based on common CBT methods such as behavioral activation and cognitive techniques. We adapted modules slightly because of the COVID-19 pandemic restrictions and provided information on the psychological consequences that were likely during the pandemic (see Appendix A). The treatment targeted psychological symptoms related to the COVID-19 pandemic and we did not present the concerns as “health anxiety”. On the other hand, we did not include much medical information (even if we had a medical doctor as part of the team), and for example refrained from giving medical advice regarding vaccination and restrictions apart from referring to guidelines by the national health authorities. This was necessary as the situation changed constantly and we could not know what the next step would be in terms of restrictions when the study was conducted. For example, schools in Sweden for small children never closed but they did so in many other countries. Given the state of knowledge it is possible that more medical information about the SARS-CoV-2, its physiological impact and how it is spread could have been included even if it was not a purpose of the study. Xiang et al. (2020) underlined that such information could be important to include when developing mental health strategies during the COVID-19 pandemic, and in particular developing psychological counseling services that are safe to provide from an infection risk perspective.

No significant correlation between number of completed modules and change between pre- and post-treatment measures was found. Thus, overall, it may seem as if symptom reduction did not depend on to which extend the participant had worked with the treatment program, as long as the individual had the treatment available. But we also need to consider the fact that the treatment was tailored to the unique participants and therefore was likely to be a good fit for the needs. Thus, potentially a smaller treatment dose might be sufficient as it was likely that the participant started to work with a prioritized problem area. In addition, it could be that being provided with a small amount of psychotherapy can be “good enough” (Barkham et al., 2006) to get an effect and that the effect does not increase when an individual continues with further modules. The phenomena of sudden gains (Shalom and Aderka, 2020), which was not explored in this study, could be a possible reason for attenuated dose-response correlations. At the same time, it is difficult to draw any conclusions as the tailored approach makes it harder to link outcome with specific modules or a specific number of modules. Furthermore, the definition of a participant having completed a module is a matter of judgement, and could instead be defined as just having a look at the module with no work involved apart from reading. This in contrast to our definition which involved actively working with the included exercises. The average completion rate of modules (approximately four out of eight) is something that should be considered in relation to the treatment content. The modules consisted mostly of text and exercises, even if for example pictures also were included. Potentially a digital treatment environment could be further improved if other elements such as quizzes or gamification features were included, as suggested by Seiferth et al. (2023). This is of importance, since a problem with the dissemination of internet treatments into clinical practice, with reduced completion rate in clinical practice compared to research studies, has been outlined. In addition, regarding the dissemination into clinical practice, this study included one year follow-up which is of importance to assess the long-term efficacy and usability of the treatment (Seiferth et al., 2023).

This study investigated an individually tailored treatment, implying that the participants received different modules in their treatment. The treatment resulted in significant effects on depression symptoms but not on posttraumatic stress for example. When looking at how many participants that had completed which modules, the modules including typical strategies aiming to target depression (Cognitive techniques and Behavioral activation) was except for the module Introduction the most commonly used. It is possible that the selection, or rather the completion, of particular modules have an effect on what symptoms that are addressed in the treatment. As mentioned, there was no significant effect found for posttraumatic stress and but relatively few participants completed that module (Difficult memories). At the same time, effects on insomnia symptoms were found even if relatively few participants had completed that module (Sleep problems). On the other hand, no effects were found on anxiety measures even if several participants had worked with the modules focusing on anxiety and exposure in different formats. Further research is needed regarding this and maybe one way to do it would be to investigate the symptom change and the specific completed modules at an individual level, still using the individually tailored approach.

Only one (2.7 %) participant in this trial was classified to be reliably deteriorated regarding depression symptoms estimated on the BDI-II (when calculating RCI). Meanwhile, there were two (5.1 %) in the control group who were classified as reliably deteriorated. Even if deterioration should be prevented, the findings are in line with the findings reported by Rozental et al. (2017), showing that the deterioration rates among 29 clinical trials of ICBT was 122 (5.8 %) for the participants in treatment and 130 (17.4 %) for the participants in control conditions. To prevent negative effects, possible deterioration should be monitored (Rozental et al., 2017). In this trial, we used the PHQ-9 as weekly measure to monitor depressive symptoms including suicidal ideation in both the treatment group and control group. More focus would, though, be needed to investigate the cases of deterioration, in particular in an attempt to detect these individuals before symptoms get worse and try to identify risk factors.

The individually tailored ICBT including weekly support by a therapist seems be able to target certain psychological symptoms, specifically depression, insomnia, and anger symptoms. A transdiagnostic approach is made possible with the individually tailoring approach, which is useful for patient populations with less distinct diagnostic boundaries (Smith et al., 2023). The results are particularly important since the trial was conducted during the COVID-19 pandemic and people seem to be affected by the pandemic in different ways. In this sense, there seems not to be a need for waiting for the (pandemic)crisis to be over to help people with psychological symptoms related to it. An essential part of COVID-19 mental health services was the home-based treatment to prevent the spread of infection (Moreno et al., 2020), which was well suited for the ICBT format. These research findings are in line with those highlighted by Smith et al. (2023) about the potential and evolving landscape of digital mental health.

### 4.1 Limitations

We mention here some limitations that need to be considered. First, a majority of the participants were female (92.1 %) and had undergone or were undergoing a college/university education (56.6 % and 32.9 % respectively). Even though this does not differ in comparison with earlier studies on internet interventions both before (Kladnitski et al., 2020; Newby et al., 2014; Titov et al., 2011) and during the COVID-19 pandemic (Brog et al., 2022; Wahlund et al., 2021), we need to be cautious with interpretations as men and less well-educated persons were underrepresented. At the same time, both women and university students were identified as vulnerable groups for developing psychiatric symptoms during the COVID-19 pandemic (Luo et al., 2020), and this could be one of the reasons why more women and more educated persons signed up for the study. However, considering the effects of ICBT on psychological symptoms and its availability, this is an area with opportunity of improvement since demographic characteristics such as gender and educational level, as well as age, marital status and having children or not, have not shown to have predictive value regarding the outcome of ICBT to date (Hedman et al., 2015).

Second, we had a relatively small sample size which limits the statistical power to detect small effects. Given that we found statistically significant treatment effects on stress symptoms as measured with the PSS-14 in the pilot study (Aminoff et al., 2021), we had expected that the results would be replicated. The results on the PSS-14 (*p* = .067, *d* = 0.43) were however in the same direction as in the pilot study. A larger sample would have generated more robust effects even if the two studies together point in the same direction. But we also need to consider the time aspect and differences between the two study samples in symptom presentation.

Third, we recruited persons affected by the COVID-19 pandemic and its consequences, regardless of if they had been infected by the virus or not. While a proportion reported that they had a confirmed test result and some reported COVID-19 associated physical symptoms, we did not check this via medical records or any test results. Much is still uncertain regarding the long-term mental and physical health consequences of being infected by COVID-19. However, Magnúsdóttir et al. (2022) reported that people with confirmed COVID-19 had more symptoms of depression and poorer sleep compared to non-infected persons in their study. The infected and non-infected people did not however differ regarding symptoms of anxiety or distress related to COVID-19 (Magnúsdóttir et al., 2022). We did not analyze our results based on whether participants had reported being infected or not. We suspected that the reliability of this reporting was not good enough, but future research on post-pandemic psychological effects will need to focus on this aspect more carefully.

## 5 Conclusions

In conclusion, this study suggests that tailored and clinician-guided ICBT can reduce psychological symptoms related to the COVID-19 pandemic. Results also indicate that symptom reductions are largely maintained one year later. Overall, the findings support the notion that tailored psychological treatments can be a way to handle uncertain situations when the symptoms experienced may be hard to predict.

## Declaration of competing interest

The authors report there are no competing interests to declare.

## Appendix A

Description of treatment modules used in the ICBT program.

***Introduction***

The introduction module was the first module for all participants. The module had focus on how mental health can be affected by pandemics and in particular the COVID-19 pandemic. CBT and CBT principles was described. Exercises included in the module were to identify the participant's own values and treatment goals.

***Behavioral activation***

Rationale for behavioral activation was given and how inactivity or negatively reinforced activities are linked to worse mood and feelings was described. Also, focus was on how activities and behaviors can be used to affect how a person feels toward a more positive and joyful way. Exercises in this module were about identifying behaviors and activities which brings enjoyable feelings and to work in different ways to increase the chance for these activities to be implemented.

***Negative thoughts***

The participants received a brief introduction to cognitive psychology and how to identify and work with negative automatic thoughts. There was a focus on how a person's interpretations can affect his or her reactions in a situation. As exercise, participants applied strategies to question their automatic thoughts and to formulate alternative and more constructive ways of thinking.

***Anxiety***

Psychoeducation about anxiety, why anxiety arises and rationale for exposure was given. Exercises was about how to create an anxiety hierarchy, plan, and work with exposure to things and situations which was included in the created hierarchy.

***Worry***

As for the module about anxiety above, this module started with psychoeducation about anxiety. It also included information about worry, different types of worry and how worry is maintained. Exercises included keeping a worry diary, implementing scheduled time for worry and postpone thoughts of worry till these specific times points.

***Panic***

The module about panic also included psychoeducation about anxiety, and furthermore about what panic is and what happens when people experience panic attacks. Rationale for interoceptive exposure was given and the participant, as exercise, got to try this through e.g., holding their breath and hyperventilate.

***Social anxiety***

Except psychoeducation about anxiety, this module focused on anxiety and fear regarding social situations. It was described how avoidance and safety behaviors maintain social situations to give rise to anxiety. The participants were given exercises to identify their safety and avoidance behaviors and to plan and work with exposure from these.

***Emotions***

Psychoeducation was given about emotions, different types of emotions, why they exist and how they can be helpful. Focus was on situations in which emotions can be experienced as problematic and how to handle these in more constructive ways. Participants got to exercise by identifying their own emotions and to distinguish between primary and secondary emotions as well as implementing strategies for managing difficult emotions.

***Acceptance***

Rationale was given about the approach of acceptance based on Acceptance and Commitment Therapy (ACT). Focus was on the paradoxical phenomena that a thought or emotion can be increasingly difficult to ignore the more the individual tries to avoid the thought or the emotion. Exercises in this module was about observing own thoughts and emotions without judging or trying to avoid them.

***Relaxation***

The rationale for applied relaxation was described. Participants get the exercise to work with the first steps of applied relaxation. The aim of the module was to give the participants a chance to feel the difference between being tense and relaxed in the body and start to pay attention to when the body is tense.

***Sleeping problems***

Psychoeducation about sleep and various factors that affect the sleep was given. Participants was introduced to strategies for managing problems of sleep. Associated exercises were about trying some of the presented strategies to ameliorate their sleep.

***Stress***

Information about stress and why it can be problematic was given. The importance and strategies for identifying and uphold a balance between demanding and recovery activities was described. Exercises consisted of identifying and creating a list of recovery activities, schedule some of them and evaluate afterwards whether they were performed or not.

***Perfectionism***

Focus within this module was unhelpful perfectionism, what consequences it can give, and self-compassion. Regarding exercises, participants got to plan and perform behavior experiment in which they challenge their assumptions about perfection. To practice self-compassion, they also get to write a letter to a friend and a letter to themselves.

***Problem-solving***

This module started with information about strategies for problem-solving. Exercises included identifying and formulating a problem present in the everyday life and around this problem list several solutions. Next thing they were encouraged to do was selecting one of these solutions to try and evaluate the result.

***Difficult memories***

Psychoeducation about how difficult experiences can affect mental health a long time after it happened was given. Rationale for imaginary exposure was also described. Participants, as exercises, was encouraged to reflect on how they are affected by their difficult experiences and memories, and they got to write about a specific memory. Also, they could list strategies to use when their memories feel overwhelming.

***Concluding module***

The concluding module, the last module for all participants, aimed to give the participants opportunity to reflect about the content of the treatment, possible rebounds and how progress can be maintained henceforth. As exercises, participants got to describe how to handle eventual rebounds, the most important things they take with them from the treatment and reflect and evaluate their treatment goals formulated previously in the introduction module.
